# Epidemiological Trends and Age-Related Outcomes in Reverse Total Shoulder Arthroplasty: A U.S. Nationwide Inpatient Analysis of 9692 Patients

**DOI:** 10.3390/jcm14238340

**Published:** 2025-11-24

**Authors:** Muhammad Khatib, Assil Mahamid, Hamza Murad, Feras Qawasmi, Eitan Lavon, Ali Yassin, Mustafa Yassin

**Affiliations:** 1 Department of Orthopedics, Hasharon Hospital, Rabin Medical Center, Tel Aviv University, Tel Aviv 6997801, Israel; 2 Gray Faculty of Medical & Health Sciences, Tel Aviv University, Tel Aviv 6997801, Israel; 3 Department of Orthopedics, Hillel Yaffe Medical Center, Hadera 3820302, Israel

**Keywords:** reverse total shoulder replacement, national inpatient sample, young patients, big data, length of stay, hospital charges

## Abstract

**Background/Objectives**: Reverse total shoulder arthroplasty (RTSA) is increasingly common, but large-scale studies on age-related differences in outcomes are limited. This study aims to evaluate epidemiological trends, clinical indications, and postoperative outcomes in patients under 65 undergoing RTSA. **Methods**: We analyzed data from the Nationwide Inpatient Sample (NIS) from 2016–2021, including 48,460 patients aged 35–64 undergoing shoulder arthroplasty. Patients were stratified into three age groups: 35–45, 46–55, and 56–65 years. We assessed trends, clinical indications, comorbidities, hospital stay metrics, and postoperative complications using chi-square tests, ANOVA, and multivariate logistic regression. **Results**: Procedure volumes increased from 2016 to 2019, peaking at 2007 cases, but declined in 2020 and 2021 (924 cases). The 56–65 age group consistently comprised the majority of procedures (81.7% in 2016 to 88.3% in 2021). Comorbidities like type 2 diabetes (5.9% to 22.8%), hypertension (27.9% to 55.3%), and dyslipidemia (10.3% to 40.5%) increased with age (*p* < 0.001). Rotator cuff tears were more prevalent in older patients (25% to 48.5%, *p* < 0.001), while instability was higher in the youngest group (8.8%, *p* < 0.001). Osteoarthritis was the most common indication, increasing with age (56.6% to 62.5%, *p* < 0.001). Cardiac complications increased with age (2.2% to 5.1%, *p* = 0.04). Female gender (OR = 1.548, *p* < 0.001), private insurance (OR = 1.570, *p* < 0.001), and small hospital size were associated with higher odds of cardiac complications. Native American race showed lower odds (OR = 0.308, *p* = 0.009) compared to White patients. **Conclusions**: Significant age-related differences exist in comorbidities, clinical indications, and postoperative complications following RTSA. Older patients had higher rates of comorbidities and cardiac complications, while younger patients presented with instability. These findings highlight the importance of age-specific perioperative management to optimize outcomes.

## 1. Introduction

Reverse total shoulder arthroplasty (RTSA) offers distinct advantages over anatomic total shoulder arthroplasty (ATSA) in select patient populations. RTSA provides superior improvements in postoperative forward elevation and abduction, particularly in individuals with preoperative limitations due to glenohumeral osteoarthritis and an intact rotator cuff [1,2]. Additionally, RTSA has been associated with lower complication and revision rates, suggesting greater long-term reliability compared with ATSA [3,4]. Patients undergoing RTSA frequently demonstrate more favorable outcomes on functional and pain assessment scales, including the Shoulder Pain and Disability Index and the American Shoulder and Elbow Surgeons score [1,3]. RTSA may also facilitate more rapid early postoperative improvements in forward elevation and external rotation [2]. Nonetheless, ATSA remains advantageous for patients with higher functional demands and preserved rotator cuff integrity, as it may yield superior external rotation outcomes [5,6]. Therefore, the selection between RTSA and ATSA should be individualized, taking into account rotator cuff status, preoperative range of motion, and patient-specific functional goals.

Several studies have substantiated the efficacy of RTSA in younger patient cohorts. Ek et al. demonstrated significant and sustained improvements in shoulder function and pain relief in patients younger than 65 years with massive, irreparable rotator cuff tears, with benefits persisting for up to a decade postoperatively [7]. Similarly, Sershon et al. reported enhanced functional outcomes in patients younger than 60 years with complex pathology, including failed rotator cuff repairs and fracture sequelae [8]. However, younger patients may experience higher complication rates relative to older cohorts. Ernstbrunner et al. documented a considerable complication burden in patients under 60 years, despite durable functional gains at long-term follow-up [9]. Neel et al. also reported inferior clinical outcomes and increased revision rates in younger individuals compared with older patients [10].

RTSA is associated with several in-hospital complications that may adversely affect outcomes. Among the most frequently reported are acromial and scapular fractures (2.5–5.4%) [7,8,9]. Glenoid loosening occurs in approximately 0.7–4.3% of cases [7,8,10], while prosthetic instability, including dislocation, has an incidence of 0.7–2.7% [7,10]. Periprosthetic fractures occur in 0.8–3.0% [11,12], and periprosthetic joint infection in 0.7–1.6% [11,12,13]. Scapular notching, unique to RTSA, is observed in 14.4% of patients [4], and neurologic injuries—particularly brachial plexus involvement—affect approximately 2% [2]. Hematoma formation, although less common, has also been reported [11,14,15].

Despite these documented associations, large-scale investigations delineating age-stratified distributions of RTSA recipients and the impact of age on postoperative complications, length of stay, hospital charges, and discharge disposition remain limited. To address this knowledge gap, the present study utilizes a nationally weighted cohort from the Nationwide Inpatient Sample (NIS), which captures approximately 20% of all U.S. inpatient discharges, spanning the years 2016–2021. By analyzing 48,460 patients younger than 65 years undergoing shoulder arthroplasty, this study aims to characterize epidemiological trends, demographic and comorbidity profiles, postoperative complication rates, hospitalization metrics, and associated healthcare expenditures. Within the constraints inherent to an administrative database, this analysis seeks to provide descriptive epidemiological insights that may support further research and inform hypothesis generation regarding perioperative risk stratification in younger RTSA patient populations.

## 2. Materials and Methods

### 2.1. Data Source

This study utilized data from the National Inpatient Sample (NIS), a large, nationally representative database maintained by the Agency for Healthcare Research and Quality (AHRQ) under the Healthcare Cost and Utilization Project (HCUP). The NIS captures approximately 20% of all inpatient discharges from hospitals affiliated with HCUP across the United States, representing an estimated seven million unweighted hospitalizations annually. By applying discharge weights provided by the NIS, these data can be extrapolated to generate national estimates, thereby offering a foundation for epidemiological and healthcare utilization research.

For this analysis, the dataset spanning 1 January 2016 to 31 December 2021 was used, reflecting the most recent data available at the time of study initiation. Each entry in the dataset, referred to as a “case,” represents a discharge-weighted sample equivalent to a cohort of approximately five patients. This weighting approach is intrinsic to the NIS’s complex survey design, ensuring that the results accurately reflect national inpatient trends.

A total of 9692 cases involving shoulder arthroplasty were identified, corresponding to a weighted population of 48,460 patients. These cases were classified into seven distinct procedural categories based on ICD-10 procedure codes, as detailed in Table 1. The majority of procedures involved synthetic prosthetic joint replacements, while a smaller subset employed advanced techniques that targeted specific joint surfaces or utilized biologic tissue substitutes.

### 2.2. Cohort Definition and Selection Criteria

The NIS database was queried for the period spanning 2016 to 2021 to identify adult patients aged 35 to 64 years who underwent shoulder arthroplasty. Patients were stratified into age-based subgroups to facilitate comparative analysis across different stages of adulthood. The age cohorts were defined as 35–45 years, 46–55 years, and 56–64 years.

A total of two procedural categories were identified, both pertaining to the implantation of synthetic prosthetic devices. The dataset included 9692 unique cases, corresponding to a nationally weighted estimate of 48,460 patients. Consistent with the NIS sampling design, each unweighted case represented approximately five discharge-weighted cases, allowing the cohort to accurately reflect national inpatient trends for shoulder arthroplasty procedures.

### 2.3. Outcome Variables (End Points)

The primary objective of this study was to evaluate trends, clinical indications, and inpatient postoperative outcomes associated with RTSA using data from the Nationwide Inpatient Sample (NIS). The analysis focused on outcomes available within the NIS, including inpatient mortality, length of hospital stay (LOS), total hospital charges, and the incidence of inpatient postoperative complications.

Postoperative complications were categorized into several domains for comprehensive assessment. Vascular complications included venous thromboembolism (VTE), encompassing both deep vein thrombosis (DVT) and pulmonary embolism (PE). Infectious complications were defined by the presence of surgical site infections. Cardiovascular events included myocardial infarction and cardiac arrest. Respiratory complications encompassed conditions such as pneumonia and respiratory failure. Finally, renal complications were assessed through the occurrence of acute kidney injury.

### 2.4. Statistical Analysis

All statistical analyses were performed using R (version 4.3.2). Missing data were handled using multivariate imputation by chained equations (MICE) with five imputations and ten iterations per dataset. The imputation model included all demographic and clinical covariates with <10% missingness, and diagnostic convergence was confirmed using trace and density plots. Outcome variables were not imputed. To generate nationally representative estimates, all analyses incorporated the NIS survey design using the survey package. Discharge weights (DISCWT), hospital clusters (HOSP_NIS), and stratification variables (NIS_STRATUM) were applied, and standard errors were calculated using Taylor-series linearization.

Categorical variables were compared across age groups using chi-square tests, while continuous variables were evaluated using one-way ANOVA. Temporal trends from 2016 to 2021 were analyzed using weighted chi-square tests.

A weighted multivariable logistic regression model was constructed only for the primary endpoint of cardiac complications. Predictor variables were selected a priori based on clinical relevance and prior literature and were included if associated with the outcome at *p* < 0.10 in univariate analyses. Multicollinearity was assessed using variance inflation factors (VIF < 3). Model diagnostics included Hosmer–Lemeshow goodness-of-fit testing, evaluation of influential observations (Cook’s distance), and model discrimination quantified using the area under the receiver operating characteristic curve (AUC).

A post hoc power analysis for the primary outcome used the weighted effective sample size, an alpha level of 0.05, and an anticipated effect size equivalent to an odds ratio of 1.20. This analysis confirmed >80% power to detect medium effect sizes in the study sample.

### 2.5. Ethical Approval and Author Disclaimer

The study was granted exempt status by the institutional review board, as the de-identified nature of the NIS dataset ensured compliance with ethical standards for human subject research. Artificial intelligence (AI)–based tools, including ChatGPT (OpenAI, GPT-5), were used solely for grammar refinement, language editing, and contextual enhancement during manuscript preparation. These tools were not used for data analysis, statistical processing, or interpretation of results, all of which were conducted independently by the research team.

## 3. Results

**Figure 1:**
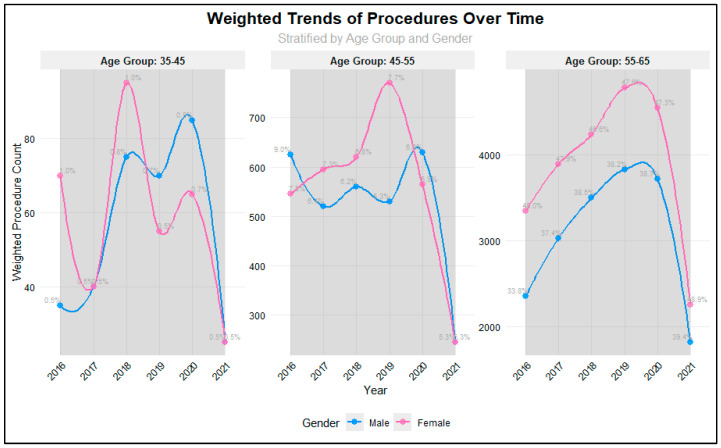
Weighted Trends in Different Age groups among RTSA patients.

Analysis of procedure volumes from 2016 to 2021 revealed significant variations across age groups and years (*p* = 0.001), as illustrated in Figure 1. The majority of procedures were consistently performed in the 56–64 age group, with proportions increasing from 81.7% in 2016 to 88.3% in 2021. The 45–55 age group showed a steady decline in representation, decreasing from 16.8% in 2016 to 10.6% in 2021, while the 35–45 age group maintained relatively stable but low proportions (ranging from 0.98% to 1.87%). Procedure volumes showed positive year-over-year growth from 2016 through 2019, with increases of 16.4% (2016–2017), 11.9% (2017–2018), and 10.4% (2018–2019). However, a notable decline began in 2020 (−4.2%), followed by a substantial decrease in 2021 (−51.9%), likely reflecting the impact of the COVID-19 pandemic. Gender analysis revealed no significant differences in procedure distribution between males and females across the study period (*p* = 0.74), suggesting that access to procedures was not influenced by gender. Total procedure volumes peaked in 2019 (*n* = 2007) before declining to their lowest point in 2021 (*n* = 924).

Our analysis included 9692 unweighted cases, corresponding to a weighted national estimate of 48,460 patients, stratified into three age groups: 35–45 years (weighted *n* = 680; 1.4%), 46–55 years (weighted *n* = 6455; 13.3%), and 56–64 years (weighted *n* = 41,325; 85.3%). Hospital characteristics were similarly distributed across all age groups, with no significant differences in bed size (*p* = 0.63), location/teaching status (*p* = 1.0), or regional distribution (*p* = 1.0). Large, urban teaching hospitals treated the highest proportion of patients across all age groups (44.0–44.1%), while the South region consistently managed the largest share of patients (38.0–38.2%). Patient demographic characteristics showed comparable distributions across age groups (Table 2). Race distribution was similar across age groups (*p* = 0.98), with White patients comprising the majority (65.0–65.4%), followed by Black (14.0%) and Hispanic (11.0%) patients. These figures reflect NIS administrative race classifications, which do not capture mixed-race identities and may vary based on hospital-reported coding. Private insurance was the predominant primary payer across all age groups (75.0%), with no significant differences in payer mix (*p* = 0.84).

However, significant age-related differences emerged in comorbidity profiles. The prevalence of type 2 diabetes increased markedly with age, from 5.9% in the 35–45 group to 22.8% in the 56–64 group (*p* < 0.001). Similar age-related increases were observed for hypertension (27.9% to 55.3%, *p* < 0.001), dyslipidaemia (10.3% to 40.5%, *p* < 0.001), and sleep apnea (8.1% to 20.4%, *p* < 0.001). Mental disorders showed a unique pattern, peaking in the middle age group (56.5% in 45–55 years) compared to both younger (49.3%) and older (47.5%) groups (*p* < 0.001). Chronic renal disease demonstrated a significant age-gradient, increasing from 1.5% in the youngest group to 6.0% in the oldest group (*p* < 0.001). Notably, certain comorbidities including anemia (*p* = 0.22), Alzheimer’s disease (*p* = 0.93), Parkinson’s disease (*p* = 0.36), and congestive heart failure (*p* = 0.29) showed no significant differences across age groups, though this may be partially attributed to their overall low prevalence in the study population (Table 3).

**Figure 2:**
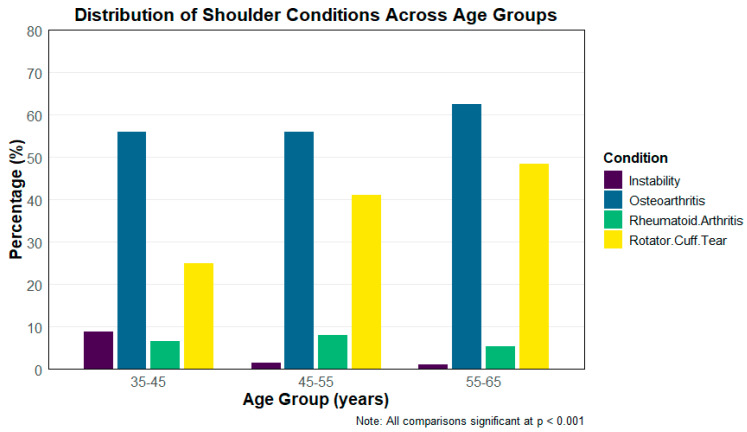
Common Indications in Different Age groups for RTSA Surgery.

The distribution of clinical characteristics among patients undergoing RTSA varied significantly across age groups (35–45 years, 46–55 years, and 56–64 years). As presented in Figure 2, rotator cuff tears were increasingly prevalent with age, affecting 25% of patients in the 35–45 age group, 41.2% in the 46–55 group, and 48.5% in the 56–64 group (*p* < 0.001). Notably, traumatic indications both acute and chronic were rare across all age groups in this cohort. This is unexpected given that trauma-related pathology is often more common among younger and more active patients, whereas osteoarthritis typically predominates in older adults. The low observed frequency likely reflects coding patterns in the NIS and the predominance of degenerative indications for RTSA during the study period. Conversely, rheumatoid arthritis was most prevalent in the 45–55 age group (8.0%) compared to 6.6% and 5.4% in the youngest and oldest groups, respectively (*p* = 0.001). Osteoarthritis was the most common indication overall, with rates increasing significantly with age: 56.6%, 56.0%, and 62.5% in the respective age groups (*p* < 0.001). Instability was more frequently observed in the youngest cohort (8.8%) compared to 1.4% and 1.1% in the older groups (*p* < 0.001). These findings highlight notable variations in the underlying indications for surgery across different age demographics (Table 4).

Analysis of hospital stay metrics revealed comparable patterns across age groups. Mean length of stay was slightly higher in the 35–45 age group (1.89 ± 1.86 days) compared to the 45–55 (1.73 ± 1.89 days) and 56–64 (1.72 ± 2.02 days) groups, though this difference was not statistically significant (*p* = 0.55). Similarly, while mean total charges were highest in the youngest age group ($92,696 ± $63,372) compared to the middle ($82,260 ± $51,836) and older ($81,576 ± $49,780) groups, this difference did not reach statistical significance (*p* = 0.12).

Postoperative complications showed varying patterns across age groups. Cardiac complications demonstrated a significant age-related increase (*p* = 0.04), affecting 2.2% of patients aged 35–45, 3.8% of those aged 45–55, and 5.1% of those aged 56–64. Although the unadjusted analysis demonstrated a significant age-related increase in cardiac complications (*p* = 0.04), this association did not remain significant after multivariable adjustment, indicating that comorbidity burden and demographic factors likely drive the observed age gradient. Surgical site infections showed an unusual distribution (*p* = 0.02), occurring in 0.7% of the youngest group and 0.1% of the oldest group, with no cases reported in the middle age group. Blood loss complications were relatively consistent across groups, affecting 8.8%, 9.0%, and 8.1% of patients in ascending age order (*p* = 0.55). Other complications showed no significant differences across age groups, including urinary tract infections (*p* = 0.45), respiratory complications (*p* = 0.48), and acute renal failure (*p* = 0.67). Notably, thromboembolic events (DVT, PE) were observed only in the oldest age group (0.2%), though this difference was not statistically significant (*p* = 0.19). The absence of certain complications in younger age groups (e.g., thromboembolism, surgical-site infection) reflects the extremely low event rates in the NIS and is consistent with the weighted national distribution. Overall, while most complications showed similar rates across age groups, the observed age-related increase in cardiac complications and the atypical distribution of surgical-site infections should be interpreted cautiously. Because complication diagnoses in the NIS rely on administrative coding, these findings may reflect coding variability or underreporting in addition to true clinical differences, yet they still highlight patterns that may merit consideration in age-specific perioperative management. In a weighted multivariate logistic regression analysis of factors associated with heart complications, age group comparisons showed varying odds ratios (45–55 years: OR = 0.726, 95% CI: 0.316–2.100; 56–64 years: OR = 1.272, 95% CI: 0.582–3.582), these differences were not statistically significant (*p* > 0.05).

Demographic factors played a significant role. Female patients had 54.8% higher odds of complications compared to males (OR = 1.548, 95% CI: 1.315–1.825, *p* < 0.001). Hospital characteristics were significant predictors. Medium and large hospital bed sizes were associated with lower odds of complications compared to small hospitals (Medium: OR = 0.756, 95% CI: 0.617–0.925, *p* = 0.007; Large: OR = 0.715, 95% CI: 0.599–0.855, *p* < 0.001). Teaching status also showed a protective effect, with urban teaching hospitals having the lowest odds of complications (OR = 0.573, 95% CI: 0.453–0.731, *p* < 0.001). Regarding payment status, patients with private insurance showed higher odds of complications (OR = 1.570, 95% CI: 1.227–2.001, *p* < 0.001) compared to other payment types. Race also emerged as a significant factor in the regression analysis, with varying odds ratios across NIS-defined racial categories. Although Native American patients were grouped within the ‘Other’ race category in Table 2 due to small sample size, they appear as a distinct category in the multivariable model and demonstrated lower odds of cardiac complications compared with White patients (OR = 0.308, 95% CI: 0.110–0.668, *p* = 0.009).

## 4. Discussion

This nationwide analysis of 48,460 patients undergoing RTSA between 2016 and 2021 revealed distinct temporal, demographic, and clinical patterns. Procedure volumes increased steadily until 2019 before declining sharply during the COVID-19 pandemic, with most surgeries performed in patients aged 55–65 years. Across hospitals, regional distributions and gender representation remained stable, suggesting equitable access to care. Older patients demonstrated significantly higher rates of diabetes, hypertension, dyslipidemia, and sleep apnea, while mental health disorders peaked in the middle-aged group. Surgical indications varied notably by age, with instability predominating among younger patients, rheumatoid arthritis peaking in midlife, and osteoarthritis and rotator cuff tears increasing with age. Despite these differences, hospital stay duration and total charges were comparable across age groups, indicating consistent perioperative efficiency. Complications were generally infrequent, though cardiac events increased significantly with age, and female sex, small hospital size, and private insurance emerged as independent predictors of higher complication risk. While the unadjusted analysis suggested higher cardiac complication rates in older patients, multivariable modeling demonstrated that age was not an independent predictor once key confounders were included. This highlights the importance of accounting for comorbidity burden and patient mix when interpreting age-related differences in postoperative risk.

Patients aged 55 to 65 years do not have the highest rates of shoulder replacement surgery; rather, the highest rates are observed in patients older than 65 years. Multiple large cohort studies and national registry analyses demonstrate that while the incidence of shoulder arthroplasty is increasing across all age groups, the most substantial growth and absolute numbers occur in those over 65 years of age [16,17,18,19]. The reasons for this age distribution are multifactorial. First, the prevalence of degenerative shoulder conditions such as primary osteoarthritis and rotator cuff arthropathy increases with age, making older adults more likely to require surgical intervention [17,20,21]. Second, RTSA, which is increasingly used for complex cases and rotator cuff pathology, is preferentially performed in older patients due to lower functional demands and higher rates of rotator cuff deficiency [16,21]. Third, younger patients (including those aged 55–65) are more likely to have complex or secondary pathologies (e.g., post-traumatic arthritis, osteonecrosis) and are at higher risk for revision surgery and complications, which may temper surgical indications in this group [22,23].

RTSA is independently associated with increased LOS and higher hospital charges compared to anatomic shoulder arthroplasty, regardless of age [23,24,25,26]. Cost analyses consistently show that RSA incurs greater index hospitalization charges than anatomic procedures, with median total hospital charges for RSA exceeding those for TSA by $1700–$3400 [25,26].

In our analysis of NIS data, length of stay (LOS) and total hospital charges demonstrated no statistically significant differences across the examined age groups, with a mean LOS of approximately 1.7 days and mean total hospitalization charges ranging from $80,000 to $90,000. Younger patients (<65 years) undergoing RSA have higher complication and revision rates, and younger age itself is associated with increased total charges and economic burden, especially in those under 50 years [10,26]. Specifically, patients under 40 years have the highest charges for both primary and revision procedures, with mean charges for primary surgery exceeding $41,000, compared to $39,000 in those aged 40–50 years [27]. This increased economic burden is driven by higher revision rates, more complex indications (e.g., post-traumatic arthritis, inflammatory arthropathy), and greater resource utilization [10,26].

While older age and comorbidities are strong predictors of extended LOS, younger patients undergoing RSA still experience higher charges, and the literature supports that younger age is a nonmodifiable risk factor for increased total charges [26,28]. However, the increase in LOS among younger RSA patients may be less pronounced than in older, comorbid populations, and is more strongly linked to the complexity of cases and revision risk.

Multivariate regression analysis identified several noteworthy predictors of postoperative complications following RTSA. Female sex emerged as an independent risk factor, with women demonstrating 55% higher odds of experiencing complications compared with men (*p* < 0.001). Although prior literature has shown that women may be at increased risk for certain RTSA-related complication including infection, instability, and periprosthetic fractures these specific events could not be evaluated in the current study because intraoperative and postoperative fractures are not reliably captured in the NIS. Therefore, the elevated complication risk observed among women in our cohort likely reflects differences in comorbidity burden, bone quality, and underlying pathology such as rotator cuff tear arthropathy and rheumatoid arthritis, which are more prevalent in female patients. These factors may contribute to the higher rates of NIS-recorded inpatient complications observed in female RTSA recipients.

Conversely, hospital characteristics such as larger bed capacity and urban teaching status were found to be protective, underscoring the advantages of institutional experience, specialized perioperative teams, and access to comprehensive multidisciplinary care. Interestingly, patients with private insurance exhibited a significantly higher likelihood of complications (*p* < 0.001), an unexpected finding that may reflect differences in case complexity, patient selection, or documentation and coding practices rather than intrinsic risk. Racial differences were modest overall, though Native American patients showed markedly lower odds of complications compared with White patients (*p* = 0.009). Multiple large database studies demonstrate that Black patients undergo reverse RTSA at lower rates than White patients, yet have increased odds of perioperative complications including acute myocardial infarction, pulmonary embolism, acute renal failure, sepsis, and surgical site infection as well as higher mortality and longer hospital stays [16,29]. Propensity-matched analyses confirm that while overall 30-day complication rates are similar between Black and White patients, Black patients have significantly higher mortality, longer operative times, and longer hospital stays after shoulder arthroplasty [29].

Rehabilitation protocols following rotator cuff tendon augmentation generally parallel those used for standard rotator cuff repair, with modifications designed to protect the augmented construct and facilitate tendon healing [27,30,31]. Management is typically staged and individualized according to tear characteristics, tissue quality, augmentation type, and patient-specific factors. Although our database does not allow evaluation of postoperative rehabilitation parameters or the impact of specific rehabilitation protocols, it remains important to summarize the latest evidence to contextualize postoperative recovery expectations. The initial phase (0–6 weeks) emphasizes sling immobilization and protection of the repair, with early passive range of motion (ROM) restricted to controlled forward elevation and external rotation, while active motion, internal rotation, extension, and overhead activities are avoided [27,30,32]. During the intermediate phase (6–12 weeks), patients progress to active-assisted and subsequently active ROM, although strengthening is generally deferred until at least 12 weeks to reduce retear risk and support tendon–bone healing [30,31,32]. The advanced phase (12–24 weeks) involves gradual introduction of isometric and isotonic strengthening of the rotator cuff and scapular stabilizers, followed by functional and sport-specific activities as tolerated [27,31]. Monitoring for postoperative stiffness is essential, and rehabilitation may be modified in patients at increased risk of adhesive capsulitis [31]. Evidence also suggests that supervised and home-based rehabilitation yields comparable outcomes in massive tear repairs, allowing flexibility in postoperative rehabilitation delivery [33].

This study has several important limitations related to the use of the NIS administrative database. First, all diagnoses, procedures, and complications are identified solely through ICD-10-CM/PCS codes, which introduces the possibility of miscoding, underreporting, and misclassification. Although we used previously published and validated code sets to identify shoulder arthroplasty procedures and postoperative complications, the NIS does not allow verification of coding accuracy at the patient level, and misclassification bias cannot be completely excluded. Second, the NIS captures only inpatient encounters and does not provide data on outpatient care, 30-day complications, readmissions, mortality after discharge, or long-term functional outcomes. As a result, complications occurring after discharge particularly infections, thromboembolic events, and readmissions may be underestimated. Third, the database lacks important clinical variables such as implant type, surgical approach, surgeon experience, perioperative protocols, and intraoperative details, all of which may influence outcomes but cannot be adjusted for in the analysis. Additionally, patient-level factors such as BMI, smoking status, medication use, and adherence to rehabilitation are unavailable and may confound observed associations. Fourth, because complications were captured only during the index hospitalization, the absence of 30-day follow-up disproportionately affects rare events and may bias comparisons between age groups. We attempted to mitigate this by restricting the analysis to complications that are reliably coded during inpatient stays and by applying consistent definitions across all cohorts, but some degree of underestimation is unavoidable. Finally, the study period includes the COVID-19 pandemic years, during which disruptions in elective surgery scheduling, healthcare access, and hospital resource allocation may have influenced procedure volumes and patient characteristics. Despite these limitations, the large weighted sample size, national representativeness, and standardized methodological approach enhance the robustness and generalizability of our findings. It is important to note that laboratory parameters could not be evaluated, as the NIS does not include blood test results, thereby precluding any assessment of the relationship between perioperative laboratory findings and postoperative prognosis in this cohort. Detailed evaluation of postoperative rehabilitation was not possible in this study because the NIS does not contain outpatient data, physical therapy metrics, or functional follow-up parameters; as such, rehabilitation-related outcomes fall beyond the scope of this inpatient-only dataset.

The present study possesses several notable strengths that enhance the validity and clinical relevance of its findings. Foremost, it represents one of the largest and most contemporary analyses of RTSA among adults younger than 65 years, using 9692 unweighted cases drawn from the National Inpatient Sample (NIS). Although the NIS provides discharge weights to generate nationally representative estimates, our analysis is based on the actual observed cases within the dataset. The use of this large, diverse, and nationally representative inpatient cohort improves the generalizability of the findings across different hospital settings, geographic regions, and patient demographics. The study also offers a comprehensive evaluation of temporal, demographic, and comorbidity-related trends, providing valuable insight into evolving patient selection and surgical indications across age groups. Furthermore, the inclusion of detailed analyses of hospital characteristics and payer status adds an important health systems perspective, highlighting institutional and socioeconomic factors associated with perioperative outcomes. The use of weighted multivariate regression models strengthened the robustness of the conclusions by controlling for potential confounders. Collectively, these methodological features strengthen the internal consistency of the observed trends; however, given the inherent limitations of the NIS and its restriction to inpatient data, the findings should be interpreted with appropriate caution and are not directly generalizable to other populations or care settings.

## 5. Conclusions

In conclusion, this large, nationally representative analysis provides a comprehensive overview of demographic, clinical, and hospital-related factors influencing RTSA outcomes among patients aged 35 to 65 years in the United States. Procedure utilization increased steadily until 2019 before sharply declining during the COVID-19 pandemic, reflecting external healthcare disruptions. Older patients demonstrated a higher burden of metabolic comorbidities, whereas surgical indications varied by age, with instability coded most frequently in the youngest cohort and degenerative conditions such as osteoarthritis and rotator cuff pathology predominating in older adults. However, the unexpectedly high proportion of osteoarthritis diagnoses in the 35–45-year age group, coupled with relatively low instability rates, should be interpreted cautiously, as they may reflect ICD-10 miscoding or the misclassification of post-traumatic or secondary degenerative changes rather than true primary osteoarthritis in this young population.

## Figures and Tables

**Table 1:** ICD-10 Diagnosis and Procedure Codes.

Code Type	Condition	ICD-10 Codes
	Reverse Total Shoulder Arthroplasty	0RRJ00Z, 0RRJ04Z
	Type 2 Diabetes	E11*
	Hypertension	I10
Diagnosis	Dyslipidemia	E78*
Diagnosis	Sleep Apnea	G473*
Diagnosis	Anemia	D64*
Diagnosis	Alcohol Abuse	F10*
Diagnosis	Mental Disorders	F*
Diagnosis	Alzheimer’s Disease	G30*
Diagnosis	Parkinson’s Disease	G20
Diagnosis	Chronic Renal Disease	N18*
Diagnosis	COPD	J44*
Diagnosis	Congestive heart failure	I50*
Diagnosis	Surgical Site Infection	T814*
Diagnosis	Urinary Tract Infection	N39*
Diagnosis	Cardiac Complications	I44*, I20*, I49*, I50*, I51*, I23*, R96*, I21*, I22*, I24*, I46*, T811
Diagnosis	Respiratory Complications	J96*, J95*, J80*, J15*, J18*
Diagnosis	Acute Renal Failure	N17*
Diagnosis	Embolism (DVT, PE)	I2602, I2609, I2692, I2699, I82401–I82429
Diagnosis	Blood Loss	D62*
Diagnosis	Fracture	S422*
Diagnosis	Instability	M2531*
Diagnosis	Osteoarthritis	M19011, M19012, M19019, M19111, M19112, M19119, M19211, M19212, M19219, M1990–M1992
Diagnosis	Rheumatoid Arthritis	M05111–M05819, M06011–M06819, M069
Diagnosis	Rotator Cuff Tear	M751, M7510–M7512, M752, M7520–M7522

**Table 2:** Patient Characteristic in Different Age groups among RTSA patients.

	Age Groups			
**Characteristic**	**35–45**	**45–55**	**56–64**	***p *** **Value**
**Sample size, *n***	**680**	**6455**	**41,325**	
**Hospital Characteristics**				
Hospital Bed Size, *n* (%)				0.63
Small	150 (22.1)	1420 (22.0)	9090 (22.0)	
Medium	230 (33.8)	2195 (34.0)	14,050 (34.0)	
Large	300 (44.1)	2840 (44.0)	18,185 (44.0)	
Hospital Location/Teaching, *n* (%)				1.0
Rural	95 (14.0)	905 (14.0)	5785 (14.0)	
Urban non-teaching	285 (41.9)	2710 (42.0)	17,355 (42.0)	
Urban Teaching	300 (44.1)	2840 (44.0)	18,185 (44.0)	
Hospital Region, *n* (%)				1.0
Northeast	145 (21.3)	1355 (21.0)	8680 (21.0)	
Midwest	155 (22.8)	1485 (23.0)	9505 (23.0)	
South	260 (38.2)	2455 (38.0)	15,705 (38.0)	
West	120 (17.7)	1160 (18.0)	7435 (18.0)	
**Patient Characteristics**				
Race, *n* (%)				0.98
White	445 (65.4)	4195 (65.0)	26,860 (65.0)	
Black	95 (14.0)	905 (14.0)	5785 (14.0)	
Hispanic	75 (11.0)	710 (11.0)	4545 (11.0)	
Asian	35 (5.1)	325 (5.0)	2070 (5.0)	
Other	30 (4.4)	320 (5.0)	2065 (5.0)	
Primary Payer, *n* (%)				0.84
Medicare	25 (3.7)	255 (3.9)	1735 (4.2)	
Medicaid	85 (12.5)	775 (12.0)	4960 (12.0)	
Private	510 (75.0)	4840 (75.0)	30,995 (75.0)	
Other	60 (8.8)	585 (9.1)	3635 (8.8)	

**Table 3:** Comorbidities in Different Age groups among RTSA patients.

	Age Groups			
**Characteristic**	**35–45**	**45–55**	**56–64**	***p *** **Value**
**Sample size, *n***	**680**	**6455**	**41,325**	
**Comorbidities, *n* (%)**				
Type 2 Diabetes	40 (5.9)	1300 (20.1)	9420 (22.8)	<0.001
Hypertension	190 (27.9)	3090 (47.9)	22,840 (55.3)	<0.001
Dyslipidemia	70 (10.3)	1760 (27.3)	16,740 (40.5)	<0.001
Sleep apnea	55 (8.1)	1045 (16.2)	8425 (20.4)	<0.001
Anemia	10 (1.5)	295 (4.6)	1885 (4.6)	0.22
Alcohol abuse	15 (2.2)	295 (4.6)	1130 (2.7)	0.001
Mental disorders	335 (49.3)	3645 (56.5)	19,645 (47.5)	<0.001
Alzheimer’s disease	0 (0.0)	5 (0.1)	25 (0.1)	0.93
Parkinson’s disease	5 (0.7)	20 (0.3)	260 (0.6)	0.36
Chronic renal disease	10 (1.5)	215 (3.3)	2500 (6.0)	<0.001
COPD	20 (2.9)	825 (12.8)	5225 (12.6)	0.003
Congestive heart failure	10 (1.5)	195 (3.0)	1465 (3.5)	0.29

**Table 4:** Postoperative Hospitalization Metrics and Complication Rates Following Reverse Total Shoulder Arthroplasty Stratified by Age Group (35–65 Years).

Characteristic	35–45	45–55	56–64	*p* Value
Sample size, *n*	680	6455	41,325	—
Hospital Stay Metrics				
Length of stay, days, mean (SD)	1.89 (1.86)	1.73 (1.89)	1.72 (2.02)	0.55
Total hospital charges, mean (SD)	92,696 (63,372)	82,260 (51,836)	81,576 (49,780)	0.12
Complications (*n*, %)				
Surgical site infection	5 (0.7%)	0 (0.0%)	40 (0.1%)	0.02
Urinary tract infection	0 (0.0%)	60 (0.9%)	430 (1.0%)	0.45
Cardiac complications	15 (2.2%)	245 (3.8%)	2105 (5.1%)	0.04
Respiratory complications	5 (0.7%)	135 (2.1%)	745 (1.8%)	0.48
Acute renal failure	5 (0.7%)	80 (1.2%)	595 (1.4%)	0.67
Embolism (DVT/PE)	0 (0.0%)	0 (0.0%)	95 (0.2%)	0.19
Blood loss	60 (8.8%)	580 (9.0%)	3340 (8.1%)	0.55

## Data Availability

Restrictions apply to the availability of these data. Data were obtained from HCUP and are available [https://hcup-us.ahrq.gov/] with the permission of HCUP.

## Abbreviations

The following abbreviations are used in this manuscript:

aTSA	Multidisciplinary Digital Publishing Institute
DVT	Deep Vein Thrombosis
HCUP	Healthcare Cost and Utilization Project
LOS	Length of stay
NIS	National Inpatient Sample
PE	Pulmonary embolism
RTSA	Reverse total shoulder arthroplasty
VTE	Venous thromboembolism
